# ED_95_ of remimazolam in nasal administration for attenuating preoperative anxiety in children

**DOI:** 10.3389/fmed.2023.1253738

**Published:** 2023-08-23

**Authors:** Xiang Long, Li-xia Wen, Hu Yang, Guo-hong Zhu, Qing-yun Zhang, Jing-jing Jiang, Yuan Gong

**Affiliations:** Institute of Anesthesiology and Critical Care Medicine, Yichang Central People's Hospital, China Three Gorges University, Yichang, China

**Keywords:** preoperative anxiety, remimazolam, children, biased coin design, nasal drip

## Abstract

**Background:**

Preoperative anxiety often prevails in children at higher levels than adults, which is a common impediment for surgeons and anesthesiologists. It is of great necessity to explore an appropriate medication to improve this situation. Remimazolam, a type of benzodiazepine drug, has been indicated for the induction and maintenance of procedural sedation in adults since 2020. To date, rare studies were reported to investigate the effect of remimazolam on children. In this study, we investigated the safety and efficacy of intranasal drops of remimazolam and tried to determine the 95% effective dose (ED_95_) of remimazolam in single intranasal administration in attenuating preoperative anxiety in children.

**Methods:**

In this study, 114 children were enrolled who underwent laparoscopic high-level inguinal hernia ligation between January 2021 and December 2022 and were divided into an early childhood children group and a pre-school children group. The biased coin design (BCD) was used to determine the target doses. A positive response was defined as the effective relief of preoperative anxiety (modified Yale Preoperative Anxiety Scale, mYPAS < 30). The initial nasal dose of remimazolam was 0.5 mg·kg^−1^ in the two groups. An increment or decrement of 0.1 mg·kg^−1^ was applied depending on the sedative responses. Isotonic regression and bootstrapping methods were used to calculate the ED_95_ and 95% confidence intervals (CIs), respectively.

**Results:**

A total of 80 children completed the study, including 40 in the early childhood group and 40 in the pre-school children group. As statistical analysis indicated, the ED_95_ of a single intranasal infusion of remimazolam for the relief of preoperative anxiety is 1.57 mg·kg^−1^ (95% CI: 1.45–1.59 mg·kg^−1^) in early childhood children and 1.09 mg·kg^−1^ (95% CI: 0.99–1.11 mg·kg^−1^) in pre-school children, and the CIs did not overlap each other.

**Conclusion:**

Remimazolam is an effective medication to relieve preoperative anxiety in children. Moreover, the ED_95_ of single nasal administration of remimazolam for effective relief of preoperative anxiety was 1.57 and 1.09 mg·kg^−1^ in early childhood children and pre-school children, respectively.

## Introduction

Preoperative anxiety often occurs in children, with an incidence of 50–75% (1). It usually occurs within 24 h before surgery, manifesting as agitation, body tension and rigidity, persistent complaining and crying, and abnormal silence. The currently accepted pathophysiological model indicates that preoperative anxiety stress-related pathophysiological changes occur at the hypothalamic–pituitary–adrenal (HPA) axis (2). Stress response leads to the release of adrenocorticotropic hormone (ACTH) and corticoids into the bloodstream as a result of activation of the HPA axis (3). Excessive preoperative anxiety often induces rapid heartbeat, hypertension, arrhythmia, and pain oversensitivity, and these disorders may last until surgeries end. Preoperative anxiety has great relevance for postoperative disorders, including unrest, enuresis, anorexia, apathy, solitarity, and insomnia, which can last as long as 6 months after operations (4–6). Therefore, anesthesiologists should pay great attention to preoperative anxiety in children.

The interposition of children's preoperative anxiety mainly includes behavior and drug intervention. Behavior interventions can effectively relieve preoperative anxiety, involving parents' presence at the induction of anesthesia (PPIA), video distraction, music therapy, storybook reading, and child preoperative education (7–10). Midazolam, dexmedetomidine, and melatonin are universal medications used to prevent preoperative anxiety. Midazolam belongs to the benzodiazepine drugs, which have anti-anxiety, sedative, hypnotic, and anterograde amnesia effects. It can be administered orally, intranasally, intravenously, and intramuscularly. In 1988, Wilton et al. first administered midazolam intranasally to attenuate preoperative anxiety in pre-school children with great success (11). In the following decades, a crowd of studies were conducted on the nasal administration of midazolam, mostly to improve preoperative anxiety and epilepsy in children. Numerous studies have demonstrated that nasal administration of midazolam is safe and reliable for relieving preoperative anxiety. A study by Wermeling et al. indicated that the elimination half-life of intranasal administration of 5 mg midazolam is up to 3.25 h (12), which suggests that some children may have drug residue after surgeries.

The new benzodiazepine remimazolam is an ester-based drug that is rapidly hydrolyzed in the body by tissue esterases into inactive metabolites (13). Yang et al. reported that an intravenous remimazolam injection of 0.2 mg·kg^−1^ at the end of surgery was effective in relieving postoperative delirium caused by sevoflurane (14). In the Phase I clinical trial, intravenous remimazolam exhibited relatively high-level clearance, a small stable volume of distribution, and short elimination half-life (15). Accordingly, remimazolam was characterized by a pharmacokinetic–pharmacodynamic profile with fast onset, fast recovery, and moderate hemodynamic side effects (16). To date, remimazolam has been widely used for induction and maintenance of anesthesia in adult surgeries, while few research studies have been performed on its application of sedation and anesthesia in children. This study aims not only to verify the safety and efficacy of intranasal drops of remimazolam in attenuating preoperative anxiety in children but also to determine the ED_95_ of remimazolam.

## Methods

A total of 114 children were enrolled who underwent laparoscopic high-level inguinal hernia ligation between January 2021 and December 2022 and were divided into the early childhood children (EC) group and the pre-school children (PC) group. The inclusion criteria are as follows: 1–6 years old; laparoscopic high ligation of hernia sac under general anesthesia; and American Society of Anesthesiologists (ASA) physical status I~II. The exclusion criteria are as follows: ASA physical status III or higher; administration of analgesic sedatives, antiemetic, or antipruritic drugs within 24 h before surgery; having a fever (38°C or higher) or cold symptoms within 24 h before surgery; history of bronchial asthma, moderate to severe sleep apnea syndrome, liver or kidney function impairment; history of obstructed airway or abnormal recovery from anesthesia; history of severe head trauma or intracranial hypertension within 6 months; history of mental or neurological diseases, chronic hypertension, and hypoalbuminemia; and history of allergy to any medication.

The children's vital indices, including non-invasive blood pressure (NIBP), HR, and blood oxygen saturation (SPO_2_), were continuously monitored at 5-min intervals by General Electric (GE) monitor. Before administration, 25 mg of remimazolam was dissolved in 0.9% normal saline (1 ml). Remimazolam was quickly dripped into the nasal cavity with a 1 ml syringe (removal needle) at the anesthetic area. The sequential dose was assigned according to the biased coin design (BCD), with an initial dose of 0.5 mg kg^−1^ for the first patient in both the early childhood children group (1–3 years) and the pre-school children group (3–6 years). In the field of pediatrics, people usually define early childhood aged 1–3 years and pre-school children aged 3–6 years.

The modified Yale Preoperative Anxiety Scale (mYPAS) is a reliable and effective tool to assess preoperative anxiety in children aged 0–12 years, and a higher value means severe anxiety (17, 18). The mYPAS measurement includes 22 items in five behavior categories, namely activity, vocalization, emotional expressivity, state of apparent arousal, and use of parent. The score ranges from 23.33 to 100, and it is generally administered at four time points, namely preoperative holding, walking to the operating room, entrance to the operating room, and introduction to the anesthesia mask.

Parents stay with the child from the time they enter the waiting area until the induction of inhalation anesthesia. In the anesthesia waiting area, children were transferred to the operating room for induction of inhalation anesthesia after 10 min of intranasal infusion of remimazolam. After anesthesia induction, the nurse established an intravenous channel. The anesthesiologist administered sufentanil (0.3 μg·kg^−1^), propofol (1.5 mg·kg^−1^), and rocuronium bromide (0.6 mg·kg^−1^) intravenously. After 2 min of the injection, endotracheal intubation was performed. Anesthesia was maintained by sevoflurane inhalation (minimum alveolar concentration, MAC range 0.7–1.0). Sevoflurane inhalation was stopped 5 min before the completion of the procedure, and oxygen flow was increased to 6 L/min.

Atropine 20 μg·kg^−1^, ephedrine 5 μg·kg^−1^, and epinephrine 10 μg·kg^−1^, as rescue medications, were available anytime for every patient. When HR was < 60 beats·min^−1^, atropine was given, and adrenaline was used if necessary. When the MAP was lower than 30% of the base value for more than 1 min, the inhalation concentration of sevoflurane was reduced, and ephedrine or epinephrine was given if necessary. When the MAP was above 30% of the baseline value and lasted for more than 1 min, clinical interventions were up to the doctor's clinical experiences.

### Biased coin design method

We used the BCD method (19) to determine the interventional dose level of remimazolam for every child in each group. First, the children were labeled with sequential numbers in each group. Then, the successive children were exposed to one of the sequential K dose levels with an initial dose of 0.5 mg·kg^−1^, the lowest dose in the current study, for both groups at the discretion of the investigators. K-ordered dose levels were chosen with a fixed increment of 0.1 mg·kg^−1^ between two adjacent levels. In the same group, if the former patient was observed to have a negative response, the latter would be treated with a dose of remimazolam increased by 0.1 mg·kg^−1^. Conversely, if a positive response was observed, the next patient in the same group received, in a random manner, either the same dose in *P* = 0.95 or the lower dose with the 0.1 mg·kg^−1^ decrement in *P* = 0.05. mYPAS < 30 was regarded as a positive response during the introduction to the anesthesia mask. It is generally accepted that the mYPAS score of >30 indicates significant preoperative anxiety (17).

### Statistical analysis

In BCD studies, drug dosage is not an independent variable and distribution is unknown, so we cannot evaluate sample size. In evaluating and comparing five target doses using a Monte Carlo simulation, Stylianou and Flournoy found that all of the estimators performed similarly in the study, nearby their equilibrium point when the sample size was 20, and the estimators became stabilized or close to stabilized when the sample size was 40. Thus, in this study, we included 40 subjects in each group (20).

The mYPAS was used to determine the remimazolam dose during mask induction. The ED_95_ of remimazolam for the relief of preoperative anxiety in children was estimated by using the isotonic regression estimator ${\hat{\mu }}_{3}$, a linearly interpolated dose between $P_{k}^{*}$ and $P_{k+1}^{*}$, bounding the probability of the effect “Γ” (0.95 in our study) (19). $P_{k}^{*}$ and $P_{k+1}^{*}$ are defined as the adjusted response probabilities at doses *x*_*k*_ and *x*_*k*+1_, respectively. They were obtained by the pool adjacent violators algorithm (PAVA) (19). The ~95% confidence interval (CI) of ED_95_ was obtained from a bias-corrected percentile method derived by bootstrapping, with a resampling size of 40, a replicate number of 2,000, and a target of Γ = 0.95, as described previously (19, 21). R software (R Foundation for Statistical Computing, Vienna, Austria; http://www.R-project.org/) was used to analyze the abovementioned data.

Descriptive statistics were used to summarize the demographic characteristics and secondary outcomes. BP and HR were compared in the same group using ANOVA for repeated measures. Two independent sample *t*-tests were used to compare BP and HR between different groups. Drug onset time, anesthesia time, operation time, extubation time, and recovery time were compared between the two groups using two independent sample *t*-tests. In all cases, *P* < 0.05 was considered to be statistically significant. We used IBM SPSS Statistics for Windows version 22 (IBM Corp., Armonk, NY, USA) for statistical analysis.

## Results

**Figure 1:**
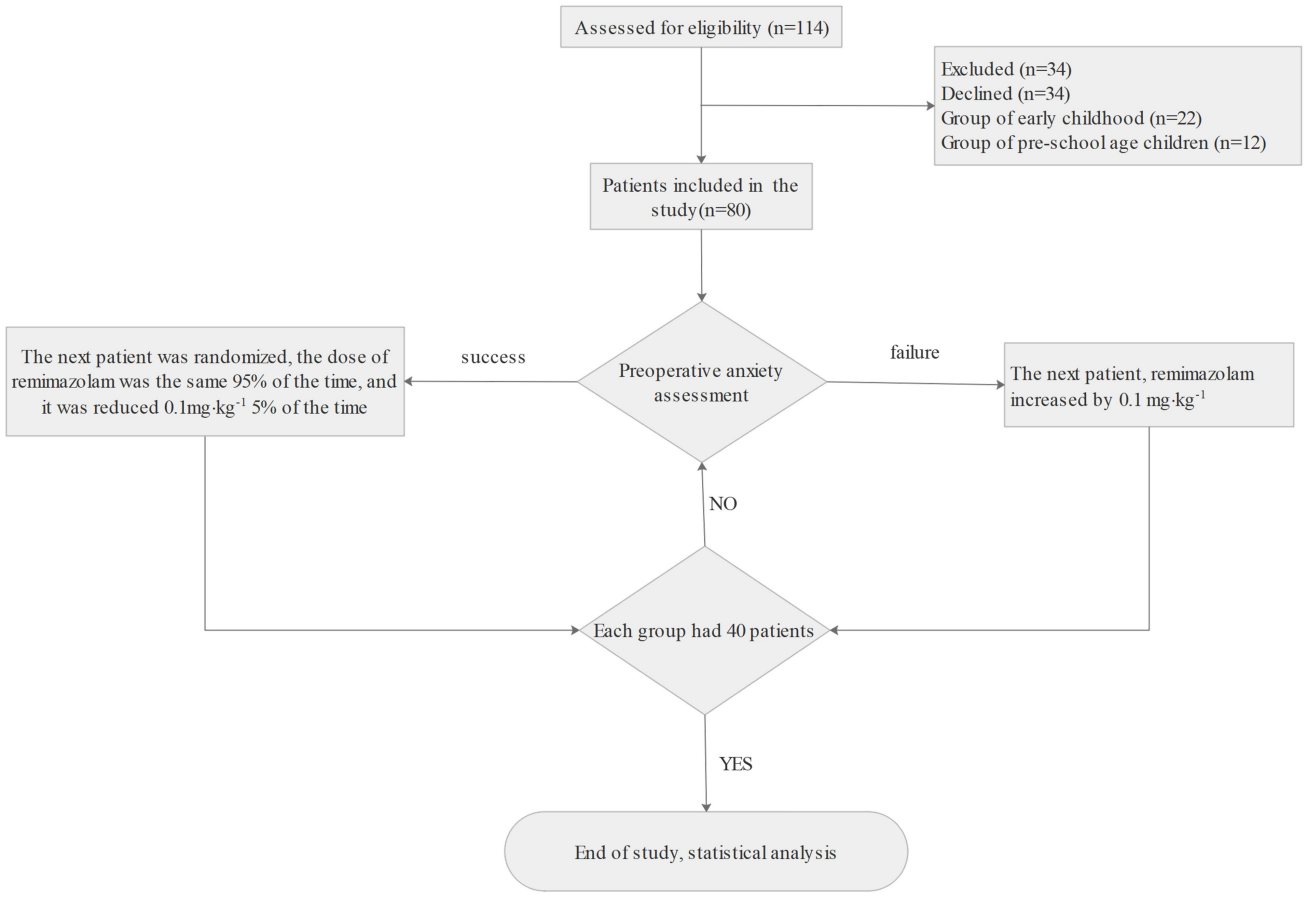
Consort flow diagram of the study.

We enrolled 114 children who underwent laparoscopic high-level inguinal hernia ligation between January 2021 and December 2022, and 80 children completed the study with ethic-informed consent, including 40 in the early childhood group and 40 in the pre-school age children group (Figure 1). The demographic characteristics of the two groups are presented in Table 1.

**Table 1:** Subject characteristics.

**Characteristics**	**Early childhood**	**Pre-school age children**
	**(*n* = 40)**	**(*n* = 40)**
Sex, male:female	33:7	31:9
**Age (months)**		
Mean [SD]	22.43 [6.17]	51.30 [10.78]
Range (months)	12.00–36.00	37.00–70.00
**Weight (kg)**		
Mean [SD]	12.21 [2.41]	17.05 [2.70]
Range	9.00–18.00	12.50–25.00
**Height (cm)**		
Mean [SD]	86.70 [8.04]	104.66 [6.94]
Range	75.00–108.00	92.00–120.00
**BMI (kg m** ^2^ **)**		
Mean [SD]	16.18 [1.78]	15.50 [1.40]
Range	13.30–20.99	13.45–20.66

**Figure 2:**
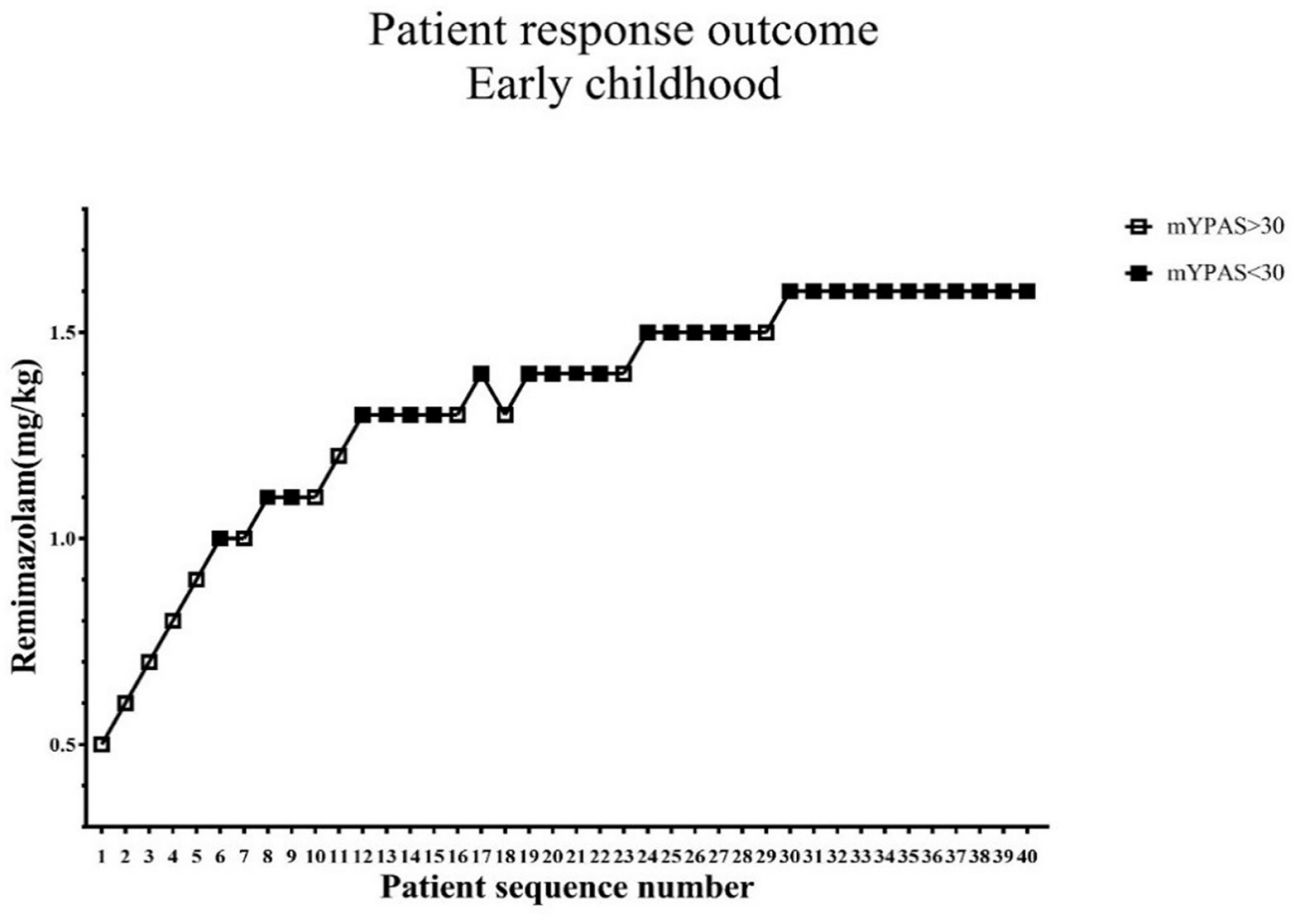
Determination of ED_95_ of single intranasal administration of remimazolam for relieving preoperative anxiety in early childhood. The subject sequence number (x-axis) is the order of subject exposures using the biased coin design. The assigned doses of remimazolam (y-axis) are 0.5, 0.6, 0.7, 0.8, 0.9, 1.0, 1.1, 1.2, 1.3, 1.4, 1.5, and 1.6 mg·kg^−1^. An effective dose (mYPAS < 30) is denoted by a solid square and an ineffective dose (mYPAS > 30) is denoted by an open square. mYPAS, modified Yale Preoperative Anxiety Scale.

**Figure 3:**
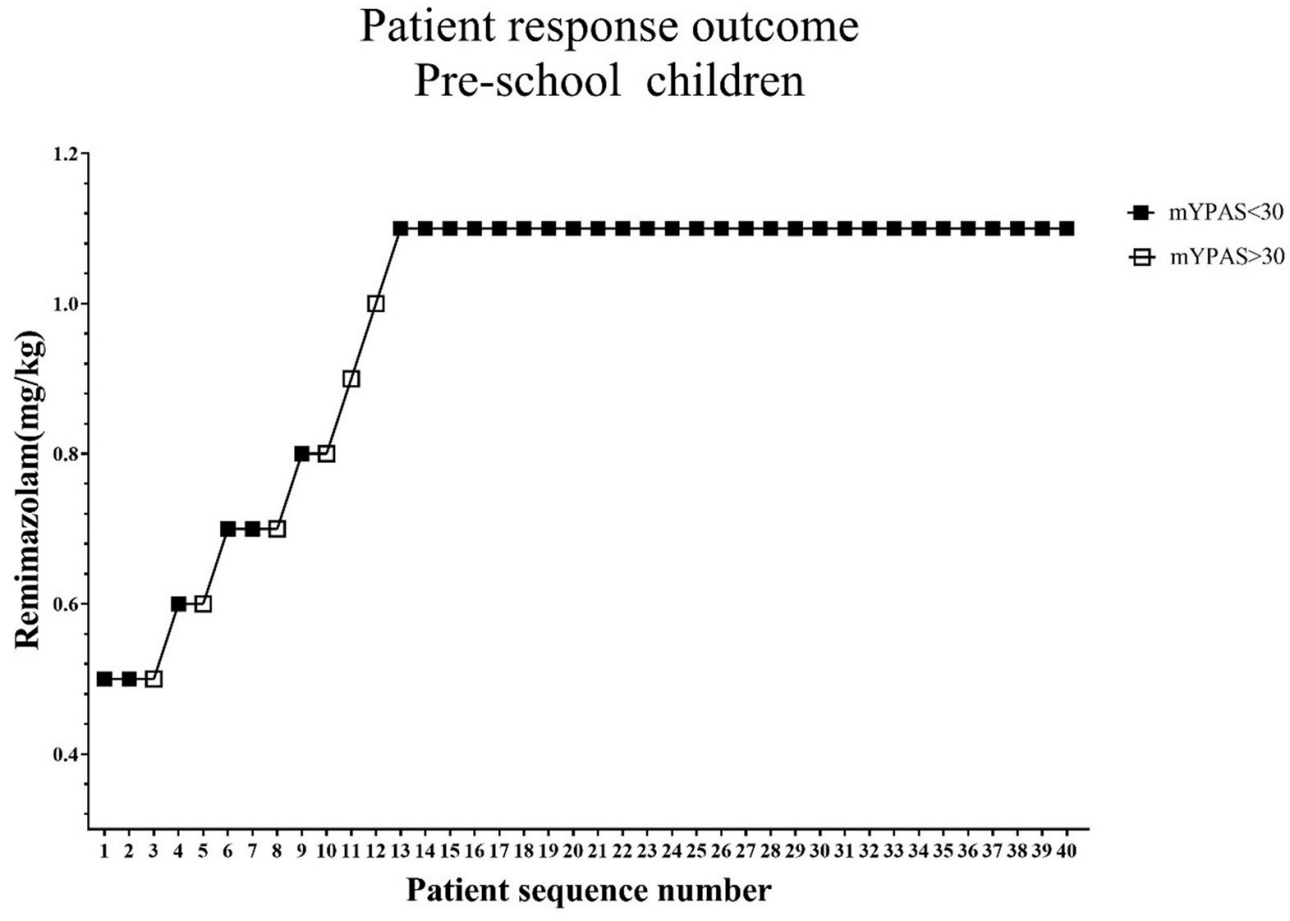
Determination of ED_95_ of single intranasal administration of remimazolam for relieving preoperative anxiety in pre-school age children. The subject sequence number (x-axis) is the order of subject exposures using the biased coin design. The assigned doses of remimazolam (y-axis) are 0.5, 0.6, 0.7, 0.8, 0.9, 1.0, and 1.1 mg·kg^−1^. An effective dose (mYPAS < 30) is denoted by a solid square and an ineffective dose (mYPAS > 30) is denoted by an open square. mYPAS, modified Yale Preoperative Anxiety Scale.

The standard chart shows the patients' effective or ineffective response at a given dose, with the sequence of children on the X-axis and the assigned dose of remimazolam on the Y-axis (Figures 2, 3). The observed response rate for each specific remimazolam dose and the PAVA-adjusted response rates are shown in Table 2. The observed response rates occasionally decreased as the dose increased, and the PAVA-adjusted response rates never decreased as the dose increased. The calculated ED_95_ is 1.57 mg·kg^−1^ (95% CI: 1.45–1.59 mg·kg^−1^) for early childhood and 1.09 mg·kg^−1^ (95% CI: 0.99–1.11 mg·kg^−1^) for pre-school children.

**Table 2:** Observed and PAVA-adjusted response rate.

**Assigned dose (mg·kg^−1^)**	**Number of successes**	**Number of tests**	**Observed response rate**	**PAVA-adjusted response rate**
**Early childhood children**				
0.5	0	1	0.000	0.000
0.6	0	1	0.000	0.000
0.7	0	1	0.000	0.000
0.8	0	1	0.000	0.000
0.9	0	1	0.000	0.000
1.0	1	2	0.500	0.500
1.1	2	3	0.667	0.500
1.2	0	1	0.000	0.500
1.3	4	6	0.667	0.667
1.4	5	6	0.833	0.883
1.5	5	6	0.833	0.883
1.6	11	11	1.000	1.000
**Pre-school children**				
0.5	2	3	0.667	0.500
0.6	1	2	0.500	0.500
0.7	2	3	0.667	0.500
0.8	1	2	0.500	0.500
0.9	0	1	0.000	0.500
1.0	0	1	0.000	0.500
1.1	28	28	1.000	1.000

**Figure 4:**
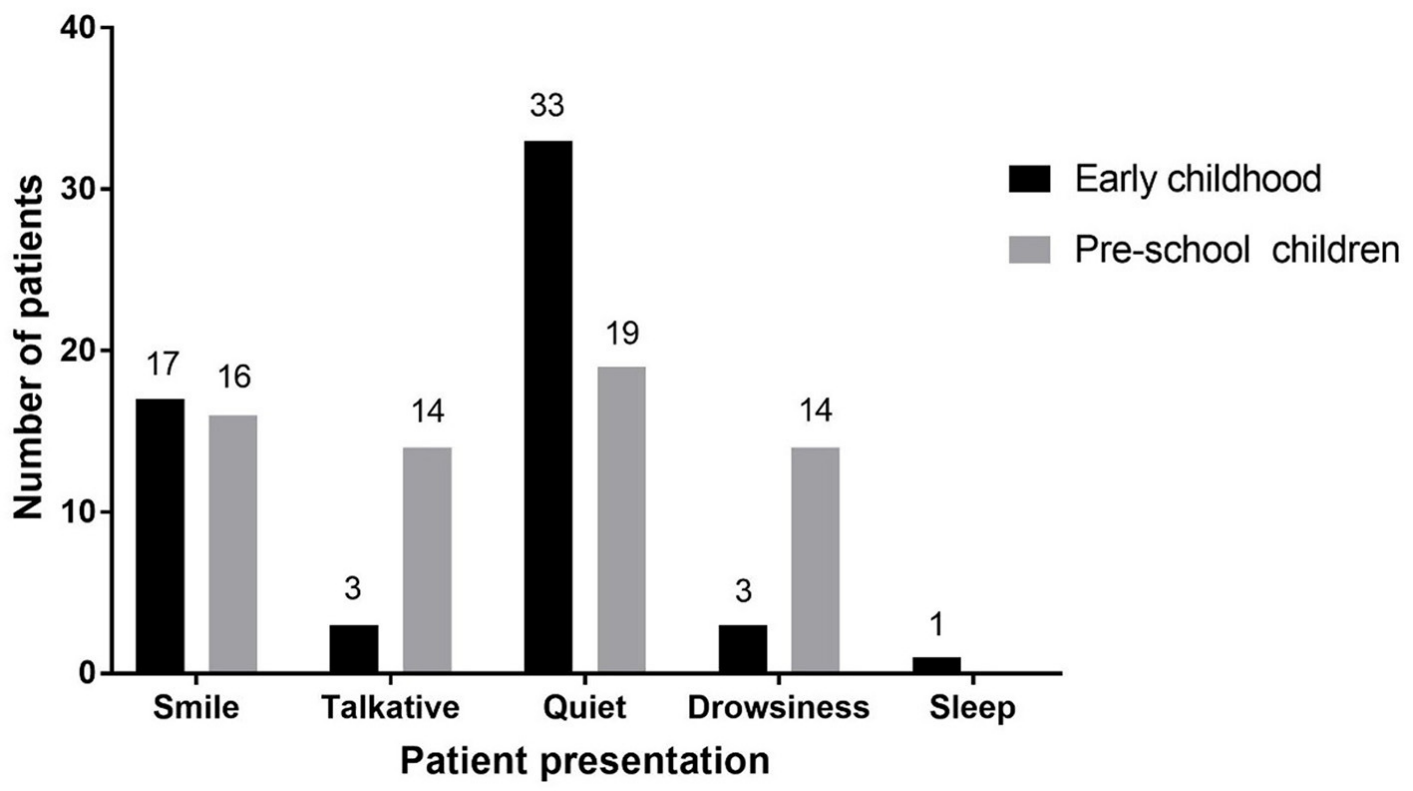
Clinical findings of patients after intranasal administration of remimazolam.

**Figure 5:**
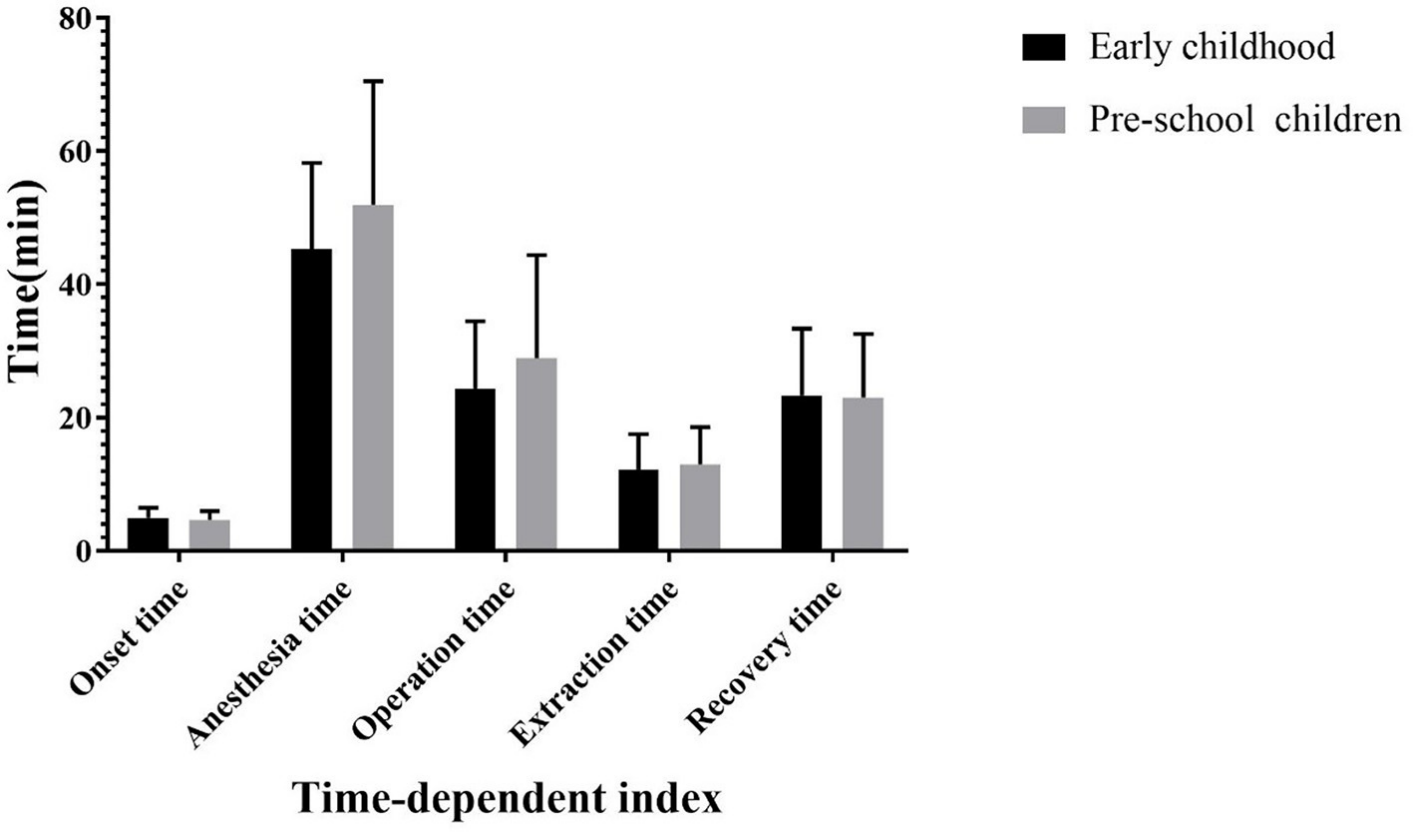
Time-dependent index during the test.

Five clinical manifestations were observed after intranasal infusion of remimazolam (Figure 4). The results of Fisher's exact test showed that there was a statistically significant difference in clinical manifestations between the two groups (*F* = 18.916, *P* = 0.000). The results of time-related indicators in the two groups, including onset time, anesthesia time, operation time, extubation time, and recovery time, are shown in Figure 5. The difference between the two groups was not statistically significant (*P* > 0.05).

**Figure 6:**
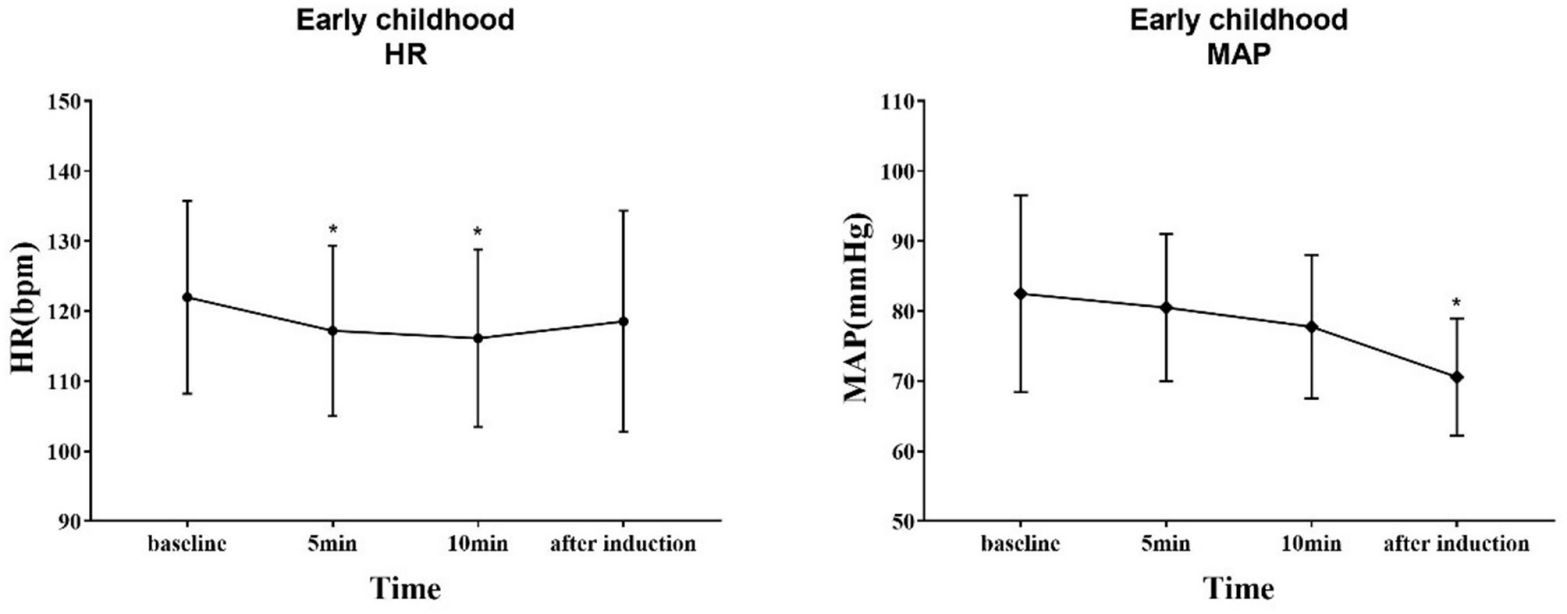
Response of HR and BP to remimazolam intranasal drip. Compared with baseline, HR decreased at 5 and 10 min after intranasal remimazolam administration (*P* < 0.05), and MAP decreased significantly after induction (*P* < 0.05). ^*^Compare with baseline (*P* < 0.05).

**Figure 7:**
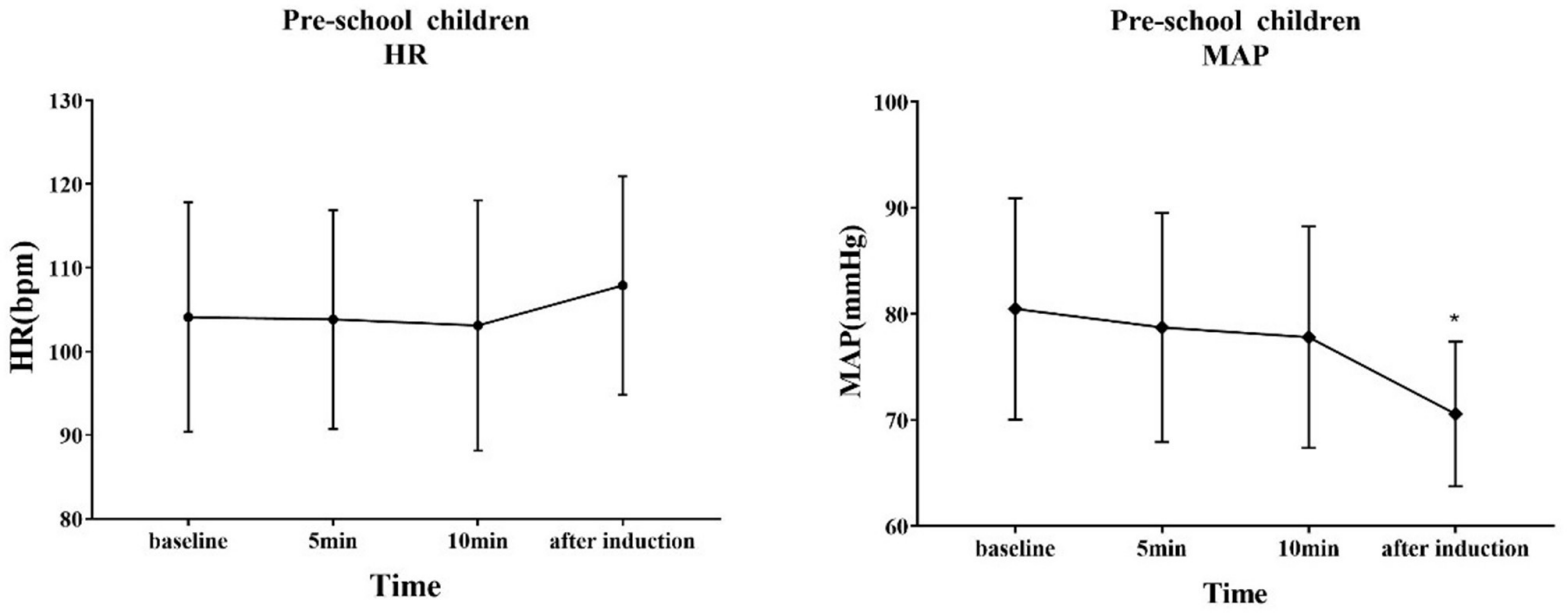
Response of HR and BP to remimazolam intranasal drip. Compared with baseline, MAP decreased significantly after induction (*P* < 0.05). ^*^Compare with baseline (*P* < 0.05).

**Figure 8:**
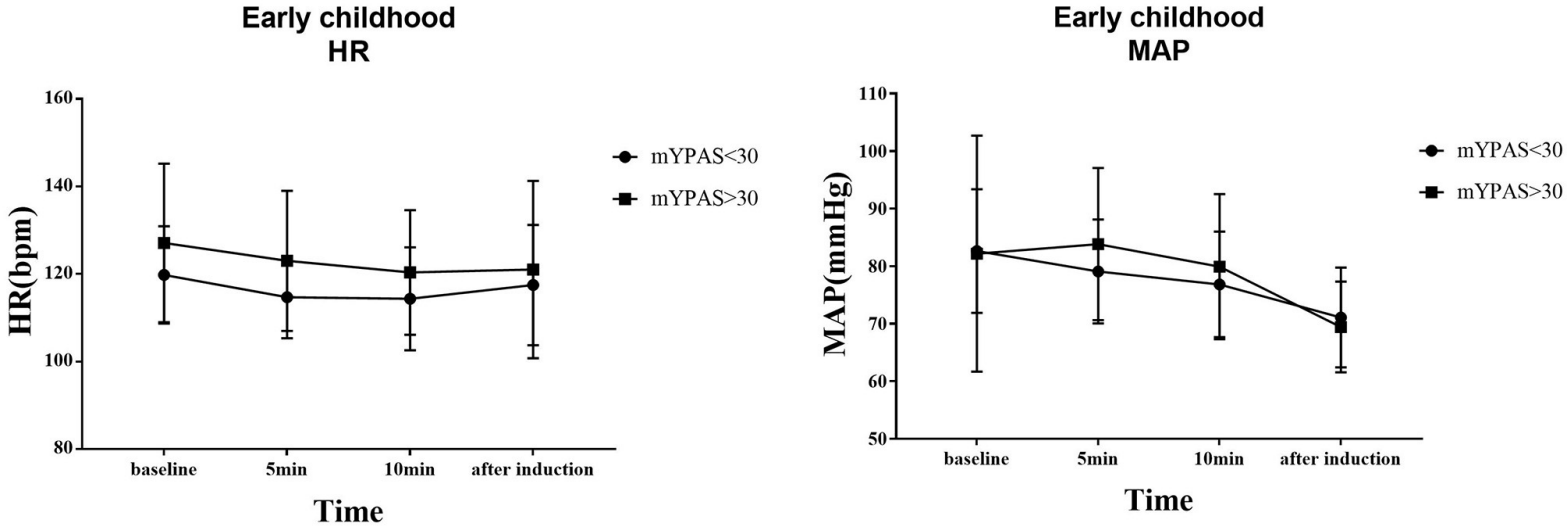
In early childhood, HR and MAP were compared between children with mYPAS < 30 and mYPAS > 30.

**Figure 9:**
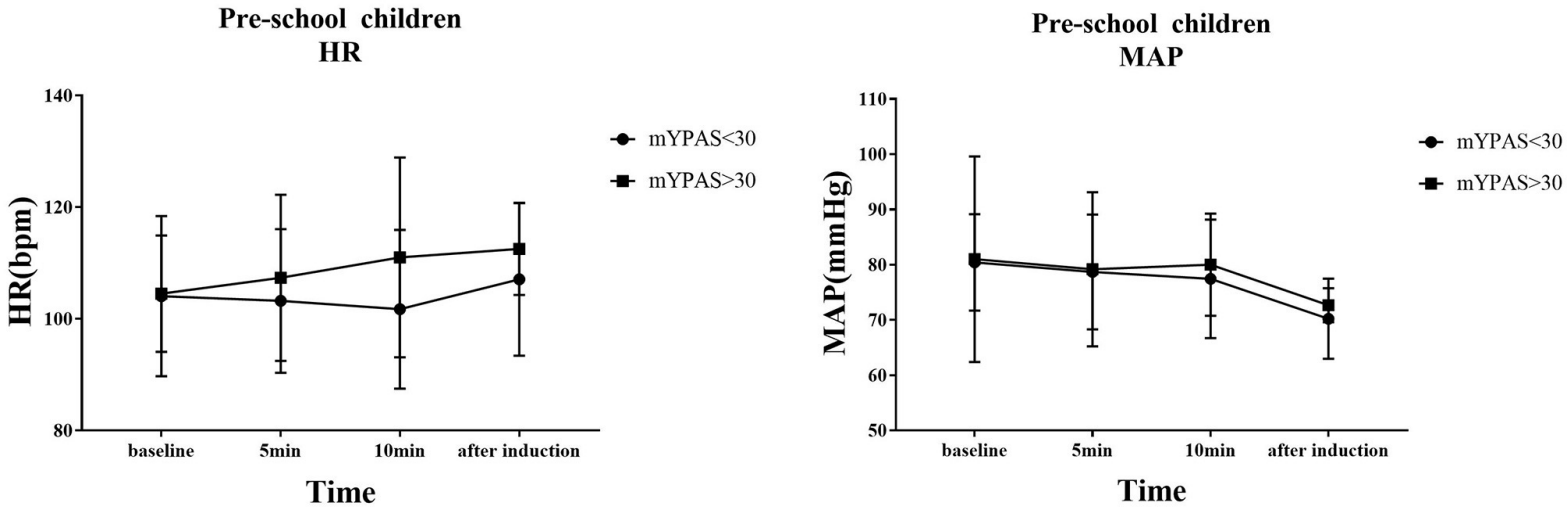
In pre-school children, HR and MAP were compared between children with mYPAS < 30 and mYPAS > 30.

The trends of HR and MAP in the waiting area and during anesthesia induction in the two groups of children are shown in Figures 6, 7. In the early childhood group, HR was lower than the baseline value after 5 and 10 min of intranasal remimazolam administration (*P* < 0.05). However, in the pre-school children group, the differences in HR at each time point were not statistically significant (*P* > 0.05). In both groups, MAP after induction was significantly lower than the baseline value (*P* < 0.05), while the difference in MAP at other time points was not statistically significant (*P* > 0.05). No decrease in MAP was more than 30% of the baseline value in each group. Comparisons of BP and HR between children with mYPAS < 30 or mYPAS > 30 in different age groups are shown in Figures 8, 9. In the same age group, there was no significant difference in MAP and HR at the same time point (*P* > 0.05).

In the anesthesia waiting area, no adverse reactions were observed, such as respiratory depression, hypoxemia, hypotension, bradycardia, nausea, vomiting, or allergy to remimazolam.

## Discussion

Remimazolam and midazolam, two benzodiazepines, share similarities in their pharmacological and pharmacokinetic characteristics, leading to common applications (22). Previous studies have clearly shown that remimazolam can be safely and effectively used for general anesthesia, for the prevention of agitation, and for sedation in pediatric magnetic resonance imaging (16, 23, 24). However, there are few reports of remimazolam for alleviating preoperative anxiety in children. Our study found that children given remimazolam through the nasal cavity did not experience any drug-related adverse reactions, and as the dosage increased, the sedative effect became better. The most important finding of this study is that the ED_95_ of a single intranasal infusion of remimazolam for relief of preoperative anxiety is 1.57 mg·kg^−1^ (95% CI: 1.45–1.59 mg·kg^−1^) in early childhood children and 1.09 mg·kg^−1^ (95% CI: 0.99–1.11 mg·kg^−1^) in pre-school children. The CIs did not overlap each other, indicating that ED_95_ was different in the two groups. Whether the degree of anxiety and characteristics of drug metabolism in different age groups contribute to this difference are unknown and need to be investigated by further research.

The BCD design method is commonly used in anesthesia studies (25–27). Binary (positive/negative) response data generated by the BCD design method are best suited by isotonic regression (28). This isotonic regression estimator has excellent statistical properties and can measure the response at any point (quantile) on the dose–response curve with little bias and variance (29). We used an isotonic regression estimator to calculate the ED_95_ of the loading dose of remimazolam on the dose–response curve.

The results of two phase IV clinical trials clearly demonstrated that effective preoperative sedation can be achieved by intranasal administration of midazolam, the classic benzodiazepine, for ~10 min (12, 30). Currently, few studies reported the pharmacokinetics of intranasal remimazolam. However, we found that the average onset time of nasal administration was 5.00 ± 1.47 min (early childhood group) and 4.68 ± 1.33 min (pre-school children group). Generally, satisfactory therapeutic effects can be achieved after 10 min of medication. It is worth mentioning that in our previous exploration, we found that the effect of remimazolam on relieving preoperative anxiety began to weaken after 20 min of administration. This finding is consistent with the pharmacokinetic and pharmacodynamic studies of intravenous remimazolam by Antonik et al. (15). Schuttle et al. found that the loss of consciousness occurred after 5 ± 1 min of intravenous remimazolam administration, and full awakening occurred after 19 ± 7 min of stopping the infusion. Thus, nasal administration of remimazolam still conforms to the pharmacodynamic characteristics of rapid onset and short action time. The extubation time and recovery time were 12.20 ± 5.32 and 23.35 ± 10.03 min in the infant group and 13.00 ± 5.55 and 23.03 ± 9.54 min in the pre-school group, respectively. The results were nearly identical in the two groups and were acceptable to anesthesiologists. However, whether preoperative medication can prolong postoperative extubation time and recovery time needs to be verified by blank controlled trials.

In the waiting area of anesthesia, the responses of children after the administration of remimazolam include smile, talkativeness, quietness, drowsiness, and sleep. In addition, the composition of children's responses to remimazolam in the two groups was different, and the main reactions of young children were smile (42.50%) and quiet (82.50%), while those of pre-school children were smile (32.50%), talkativeness (35.00%), quietness (47.50%), and sleep (35.00%). This difference may be caused by the different degrees of neurological development in children. Pre-school children have higher language ability and a higher desire for expression. The use of remimazolam during procedural sedation for colonoscopy in adults does not require mechanical ventilation, suggesting that remimazolam has little influence on respiratory function (31), which is also observed in children. Among all the children, only one manifested sleep state, and no child with excessive sedation, respiratory depression, and hypoxemia was observed. Remimazolam not only has little effect on respiration but also has a slight effect on hemodynamics (15). Our study found that the fluctuation of HR and MAP in the two groups before induction was not large compared with the baseline. Only the MAP decreased significantly after induction, but the decrease was not more than 20% of the baseline value, which was generally acceptable. The decrease in MAP after induction was mainly caused by induction drugs, such as sufentanil and propofol. In the same group, although there were no significant differences in MAP and HR at the same time points between children successfully sedated or not, the mean values of MAP and HR in children successfully sedated were still lower than those failed sedation after 5 or 10 min of nasal instillation of remimazolam. This may be due to the inadequate sample size (sedation failure, 12 in early childhood and 6 in pre-school children). Although adverse reactions such as respiratory depression, hypoxemia, hypotension, bradycardia, nausea, vomiting, and allergy were not observed, the decreased behavior ability of children may lead to accidental injuries such as tumbles. Therefore, nursing should be strengthened after nasal administration of remimazolam to prevent accidents.

## Conclusion

The intranasal administration of remimazolam is safe and effective in relieving preoperative anxiety in children. The ED_95_ of a single nasal drip of remimazolam for relieving preoperative anxiety is 1.57 mg·kg^−1^ in early childhood children and that of pre-school children is 1.09 mg·kg^−1^.

## Data availability statement

The raw data supporting the conclusions of this article will be made available by the authors, without undue reservation.

## Ethics statement

The studies involving humans were approved by the Institutional Review Board of the First Affiliated Hospital of Three Gorges University. The studies were conducted in accordance with the local legislation and institutional requirements. Written informed consent for participation in this study was provided by the participants' legal guardians/next of kin.

## Author contributions

XL: Investigation, Writing—original draft. L-xW: Writing—review and editing, Formal analysis. HY: Data curation, Formal analysis, Writing—original draft. G-hZ: Funding acquisition, Project administration, Writing—review and editing. Q-yZ: Investigation, Conceptualization, Writing—original draft. J-jJ: Project administration, Investigation, Writing—review and editing. YG: Writing—review and editing, Supervision, Validation, Visualization.
